# Supplementary material for: The interactome of intact mitochondria by cross-linking mass spectrometry provides evidence for coexisting respiratory supercomplexes
Source: Mol Cell Proteomics. 2017 Dec 8;17(2):216–32. doi: 10.1074/mcp.RA117.000470 (PMC5795388; doi:10.1074/mcp.RA117.000470)
Supplement: Supplemental Data [file supp_RA117.000470_133922_0_supp_23978_fzffwf.zip › spectra_annotation/mito_DR_spectra_annotation/473-1-5-1-1-1.pdf]

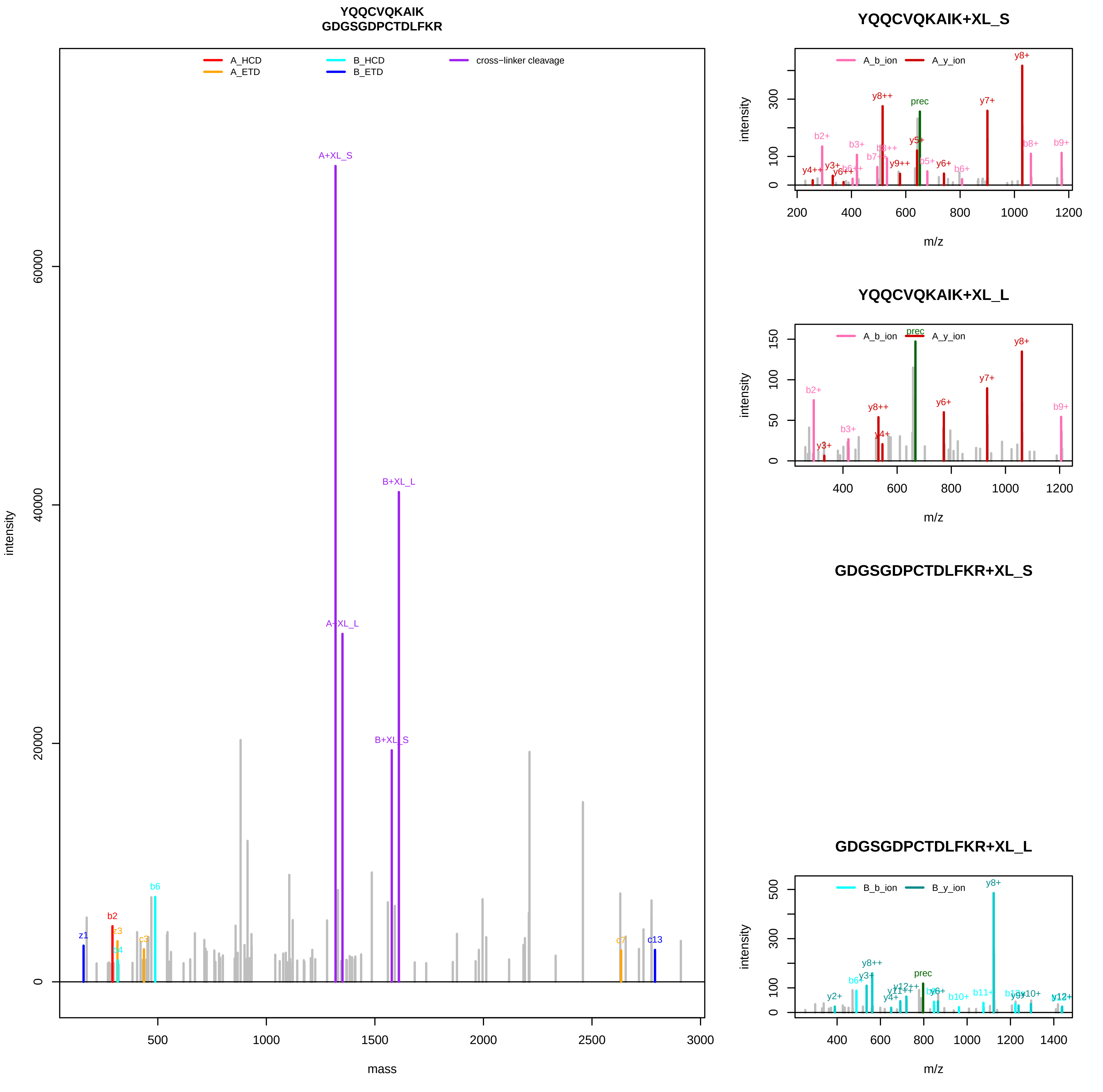

YQQCVQKAIK
GDGSGDPCTDLFKR
A_HCD
A_ETD
B_HCD
B_ETD
cross−linker cleavage
A+XL_S
B+XL_L
A+XL_L
B+XL_S
z1
b2
z3
b4
c3
b6
c7
c13
intensity
mass
0
20000
40000
60000
500
1000
1500
2000
2500
3000
YQQCVQKAIK+XL_S
A_b_ion
A_y_ion
y8+
y8++
prec
y7+
b2+
b3+
y5+
y4++
y3+
y6++
b7++
b8++
y9++
b5+
y6+
b6+
b8+
b9+
m/z
YQQCVQKAIK+XL_L
y4+
GDGSGDPCTDLFKR+XL_S
GDGSGDPCTDLFKR+XL_L
B_b_ion
B_y_ion
y8++
y3+
b6+
y2+
y11++
y12++
b10+
b11+
y10+
y12+
